# The Impact of Lateral Extra-Articular Tenodesis on Knee Range of Motion After Pediatric Anterior Cruciate Ligament Reconstruction

**DOI:** 10.1177/23259671261467539

**Published:** 2026-09-17

**Authors:** Christopher D. Hamad, Kole Joachim, Esther Chung, Shannon Wu, Timothy Liu, Mauricio Silva, Richard Bowen, Soroush Baghdadi

**Affiliations:** * Department of Orthopaedic Surgery, University of California Los Angeles, Los Angeles, California, USA; † David Geffen School of Medicine at University of California Los Angeles, Los Angeles, California, USA; ‡ The University of California Los Angeles, Los Angeles, California, USA; § Luskin Orthopaedic Institute for Children, Los Angeles, California, USA; Investigation performed at The University of California Los Angeles, Los Angeles, California, USA

**Keywords:** knee stiffness, lateral extra-articular tenodesis, pediatric anterior cruciate ligament reconstruction, postoperative range of motion, procedural arthrofibrosis, rehabilitation, risk factors

## Abstract

**Background:**

Lateral extra-articular tenodesis (LET) is increasingly used to augment anterior cruciate ligament (ACL) reconstruction (ACLR) in pediatric patients to improve graft survival and rotational stability. However, concerns remain regarding postoperative knee stiffness after augmentation.

**Purpose:**

To determine whether LET augmentation is associated with increased risk of restricted knee range of motion (ROM) after pediatric ACLR and to identify patient and surgical factors associated with postoperative stiffness.

**Study Design:**

Case-control study; Level of evidence, 3.

**Methods:**

The authors reviewed 412 pediatric patients who underwent primary ACLR (2013-2025) at a single tertiary referral center. Restricted knee ROM was defined as a loss of flexion ≥15° or extension ≥5° at 3 months postoperatively compared with the contralateral limb. Exposures included LET utilization, demographic factors, socioeconomic measures (Area Deprivation Index and insurance status), graft characteristics, meniscal procedures, time to initiation of physical therapy, and time from injury to surgery. Multivariable logistic regression models were used to identify independent predictors of restricted knee ROM.

**Results:**

Restricted knee ROM occurred in 12.9% of procedures overall (16.8% with LET vs 10.2% without). LET was independently associated with increased odds of restricted motion (adjusted odds ratio [AOR] 2 [95% CI, 1.01-3.95]; *P* = .046). Female sex was associated with increased risk (AOR, 4.23 [95% CI, 2.09-8.55]; *P* < .001). Age at surgery, time to initiation of physical therapy, graft type, meniscal procedures, socioeconomic deprivation, insurance status, and time from injury to surgery were not independently associated with restricted knee ROM. Seven patients underwent manipulation under anesthesia, and 3 underwent lysis of adhesions. Overall, manipulation under anesthesia or lysis of adhesions was required in 8 patients and was more common after LET.

**Conclusion:**

LET augmentation during pediatric ACLR is associated with an increased risk of early restricted knee ROM, although most cases are mild and resolve with nonoperative management. Female sex represented an additional risk factor for postoperative stiffness.

Anterior cruciate ligament (ACL) ruptures account for approximately 50% of all knee ligament injuries, with an overall incidence of 68.6 per 100,000 people per year.^23,29^ ACL reconstruction (ACLR) surgery is among the most common orthopaedic procedures, with approximately 200,000 ACLR procedures occurring in the United States annually.^3^ The rate of pediatric and adolescent ACLR has substantially increased over time, and reconstruction has been utilized up to 29 times more frequently in recent decades.^4,17,24,32^ This is most reflective of growing sports participation, early sports specialization, and year-round training.^1,10,18^ In addition, graft rerupture is not uncommon, and by 4 years, the rate reaches 19.7% in a large series of pediatric ACLs.^8^ These graft-failure concerns have contributed to increasing use of lateral extra-articular augmentation procedures (LEAPs) in high-risk young athletes. However, because these procedures add extra-articular restraint to the reconstructed knee, their expanding use has also renewed interest in whether improved graft protection may come at the cost of early postoperative stiffness or delayed recovery of knee range of motion (ROM).

To improve graft survival rates, LEAPs have grown in popularity in the last decade. LEAPs are often added in high-risk patients, including patients with ligamentous laxity, high-grade pivot shift, and revision cases, and are thought to improve the anterolateral rotatory stability of the knee.^15,16,21^ Multiple LEAP techniques have been described, including anatomic anterolateral ligament reconstruction and nonanatomic lateral extra-articular tenodesis (LET) procedures using the iliotibial band. Among these, LET techniques derived from the Lemaire procedure have been widely adopted, typically involving passage of an iliotibial band strip deep to the lateral collateral ligament with femoral fixation near the lateral epicondyle.^14,21,31^ More directly relevant pediatric and adolescent data have also supported LET augmentation in high-risk young patients. In a comparative cohort of 111 early adolescent patients, Monaco et al^22^ reported a significantly lower graft rupture rate after ACLR combined with LET compared with isolated ACLR using hamstring tendon autograft, with no graft ruptures in the ACLR+LET group versus a 15% rupture rate after isolated ACLR. The combined group also demonstrated improved pivot-shift stability, reduced side-to-side anterior laxity, higher Tegner activity scores, and no increase in nongraft rupture-related reoperations or complications. Carrozzo et al^5^ systematically reviewed the combined use of ACLR and LET in skeletally immature patients. They reported an overall graft failure rate of 4.65%, a return-to-play rate of 95.11%, and low rates of coronal plane deformity and limb-length discrepancy.

Despite a successful reduction in graft rerupture rates, biomechanical studies suggest that some lateral extra-articular procedures may overconstrain the knee, limiting internal tibial rotation and increasing lateral compartment contact pressures, particularly in deep flexion. Importantly, available clinical follow-up studies have not consistently demonstrated corresponding reductions in ROM or increased radiographic osteoarthritis after LEAP augmentation. Viglietta et al^33^ reported long-term clinical and radiographic outcomes after ACLR combined with the Arnold-Kocher modification of the MacIntosh LET. They found that the addition of LET did not appear to increase the risk of radiographic osteoarthritis.^33^ Zaffagnini et al^35^ similarly reported durable outcomes after over-the-top ACLR with hamstring tendon autograft and extra-articular lateral tenodesis at a minimum 20-year follow-up, including good laxity control and no significant lateral or patellofemoral joint-space narrowing, concluding that the lateral extra-articular plasty did not generate lateral or patellofemoral osteoarthritis.^35^ Nevertheless, whether LET contributes to early postoperative motion restriction remains clinically relevant, particularly as LET use expands in pediatric and adolescent patients. A multicenter French Arthroscopy Society study including 392 patients undergoing ACLR with lateral extra-articular techniques reported a 1.3% rate of arthrofibrosis requiring lysis of adhesion (LOA) or manipulation under anesthesia (MUA) and a 1.7% complication rate attributable to LEAPs. However, this estimate reflects a procedural definition that captures more severe or refractory stiffness rather than the broader spectrum of postoperative motion loss.^27^ In addition, a recent single-surgeon study reported no increased risk of arthrofibrosis after adolescent ACLR with LET augmentation. However, that series was limited to hamstring tendon autograft reconstructions and therefore may not fully reflect contemporary pediatric graft heterogeneity, which includes quadriceps tendon and bone-patellar tendon-bone (BPTB) autograft use in addition to hamstring tendon autograft.^7,20^

Therefore, this study aimed to review a single-center institutional cohort of pediatric patients undergoing ACLR with and without LET augmentation to determine whether LET was associated with 3-month postoperative restricted knee ROM. Secondary aims included evaluating the effects of time to physical therapy initiation, socioeconomic status, graft type, time from injury to surgery, and other surgical factors on restricted ROM, as well as comparing rates of procedural arthrofibrosis requiring MUA or LOA. We hypothesized that LET augmentation would be associated with increased risk of restricted knee ROM.

## Methods

### Data Source and Variable Selection

We conducted a retrospective cohort study at a single institution between 2013 and 2025. The primary outcome was 3-month postoperative restricted ROM, defined as an extension deficit of ≥5° or a flexion deficit of ≥15° compared with the contralateral knee. Postoperative ROM was obtained from routine clinical follow-up documentation at the 3-month postoperative visit and was based on the treating clinician’s recorded examination. These thresholds were selected based on previous ACLR literature that used side-to-side ROM differences to identify postoperative motion loss.^6^ Since the endpoint was defined relative to each patient’s contralateral knee, physiologic hyperextension was considered part of native terminal extension. Accordingly, loss of terminal hyperextension was retained when it resulted in a ≥5° side-to-side extension deficit. Patient factors and time to physical therapy (measured categorically as <14 days, 14 to 30 days, and >30 days from ACLR) were assessed for restricted ROM risk, as well as the occurrence of MUA or LOA as secondary outcomes. Pediatric patients (age at the time of surgery <18 years) who underwent primary ACLR with quadriceps, hamstring tendon, or BPTB autografts were included in the analysis. Patients were excluded if they had multiligamentous injuries, revision surgery, allograft tendon use, or incomplete operative or follow-up data. Adequate follow-up for the primary endpoint was defined as postoperative follow-up at or >90 days with sufficient ROM documentation to determine restricted knee ROM. Exclusion for inadequate follow-up was compared between the LET and non-LET groups using chi-square testing. Data collected included patient characteristics, insurance type, time from injury to surgery, Area Deprivation Index (ADI) as a measure of socioeconomic status, surgical details (graft diameter, radiographic notch width, use of LET, graft type, meniscal treatment), postoperative recovery metrics (ROM at 3 months, time to first physical therapy appointment), and postoperative complications (cyclops lesion, need for MUA, or LOA). Cyclops lesions were analyzed separately and were not included in the primary restricted-motion outcome. The notch width was measured retrospectively from available preoperative magnetic resonance imaging (MRI) on the axial image demonstrating the intercondylar notch. The measurement was obtained as the transverse distance between the medial and lateral femoral condylar margins at the notch, using bicondylar width as an anatomic reference for orientation when available. Patients with postoperative motion deficits were initially managed with supervised rehabilitation and observation. At the 3-month postoperative visit, patients with persistent flexion or extension deficits despite therapy were identified as having restricted postoperative ROM. However, procedural intervention was reserved for patients with persistent, functionally limiting stiffness that failed to improve with continued rehabilitation or when a mechanical block to motion was suspected. MUA was generally performed first in cases of persistent stiffness without a clear mechanical block. If full motion was restored after MUA, no additional procedure was performed. LOA was reserved for patients with residual motion loss after MUA or with MRI-confirmed cyclops lesions, suggesting a mechanical limitation of motion.

### Surgical Technique

Two fellowship-trained pediatric sports medicine surgeons (R.B., S.B.) performed procedures at a single tertiary referral center. ACLR was performed arthroscopically using quadriceps tendon, hamstring tendon, or BPTB autograft. Graft selection was determined by the treating surgeon based on patient age, skeletal maturity, sport demands, graft availability, anatomic considerations, and surgeon preference, and evolved over the study period. Earlier in the study period, hamstring tendon autograft was used more commonly, with BPTB autograft used in select patients. Later in the study period, graft selection shifted toward predominant use of quadriceps tendon autograft. BPTB autograft was generally reserved for select circumstances, including revision cases, patients with previous contralateral BPTB autograft, or patients who selected BPTB after discussion of graft options. Tunnel technique and fixation construct were similarly individualized according to skeletal maturity, graft choice, and surgeon preference. ACL fixation strategy was surgeon-dependent, with some surgeons preferentially using interference screw fixation and others preferentially using suspensory fixation. Femoral and tibial sockets/tunnels were prepared arthroscopically, grafts were cycled, and ACL grafts were tensioned in full extension before final fixation. Graft position and absence of notch impingement were confirmed arthroscopically in full extension.

LET indications also evolved during the study period. Initially, LET was performed primarily in patients considered at elevated risk for graft failure or residual rotatory instability, including revision-risk cases and patients with preoperative high-grade pivot shift. After 2023, LET was performed routinely as part of institutional practice for pediatric and adolescent ACLR. When LET was performed, it was generally completed as an iliotibial band–based modified Lemaire-type tenodesis. A central strip of the iliotibial band was harvested while maintaining distal attachment at the Gerdy tubercle, passed deep to the lateral collateral ligament from anterior to posterior, and fixed on the femur at or near a point proximal and posterior to the lateral epicondyle. The same modified Lemaire-type LET was used by all participating surgeons throughout the study period. LET femoral fixation varied by surgeon and operative era, with fixation evolving from interference screw fixation to knotless all-suture anchor fixation around 2024. LET fixation was performed with the knee in 30° to 45° of flexion and neutral rotation.

At the conclusion of surgery, all patients were allowed weightbearing as tolerated and were placed in a hinged knee brace locked in extension while sleeping and ambulating. ROM exercises were initiated on postoperative day 1, with the brace unlocked to allow motion from 0° to 90°. Patients and families received postoperative instructions regarding brace use, weightbearing progression, early home exercises, and formal physical therapy initiation. Formal physical therapy was prescribed postoperatively for all patients, typically to begin within the first 2 postoperative weeks. Patients were followed at routine postoperative intervals to monitor wound healing, pain control, ROM recovery, and progression through rehabilitation. The postoperative rehabilitation protocol was the same before and after LET was added to institutional practice, and ROM progression was not intentionally delayed or restricted because of LET augmentation alone.

### Statistical Analysis

Descriptive statistics were used to summarize patient characteristics and outcomes. Continuous variables are presented as means with standard deviations or medians with interquartile ranges (IQR), as appropriate, and categorical variables as counts with percentages. Parametric continuous variables were analyzed using *t* tests, and nonparametric continuous variables were analyzed using the Wilcoxon rank-sum test after assessment of normality using the Shapiro-Wilk test. Associations between the ADI and categorical time-to-physical-therapy groups were assessed using 1-way analysis of variance with Bonferroni-adjusted pairwise comparisons. Categorical variables were compared using chi-square tests. Adjusted odds ratios (AORs) with 95% CIs were reported from multivariable regression analyses. *P* values < .05 were considered statistically significant. All analyses were performed using Stata (StataCorp). The study was approved by the institutional review board and was conducted in compliance with the Health Insurance Portability and Accountability Act.

## Results

### Patient Characteristics

The final analytic cohort included 412 procedures after the application of exclusionary criteria (Figure 1). Of those, 199 (48.3%) were female with a mean age at time of surgery of 16.3 ± 1.4 years. The median duration of follow-up was 380 days (IQR, 251-810 days). Age at surgery differed modestly between sexes (female, 16.2 ± 1.4; male, 16.5 ± 1.4 years; *P* = .028). Most patients were insured through Medicaid or affiliated plans (73.1%; n = 301), compared with commercial plans (26.9%; n = 111). The median state-level ADI decile was 6 (IQR, 4-7), with a mean of 5.4 ± 2.3, while the median national ADI percentile was 15 (IQR, 8-21), with a mean of 15.9 ± 10.7. Complete patient characteristics are summarized in Table 1.

**Figure 1.**
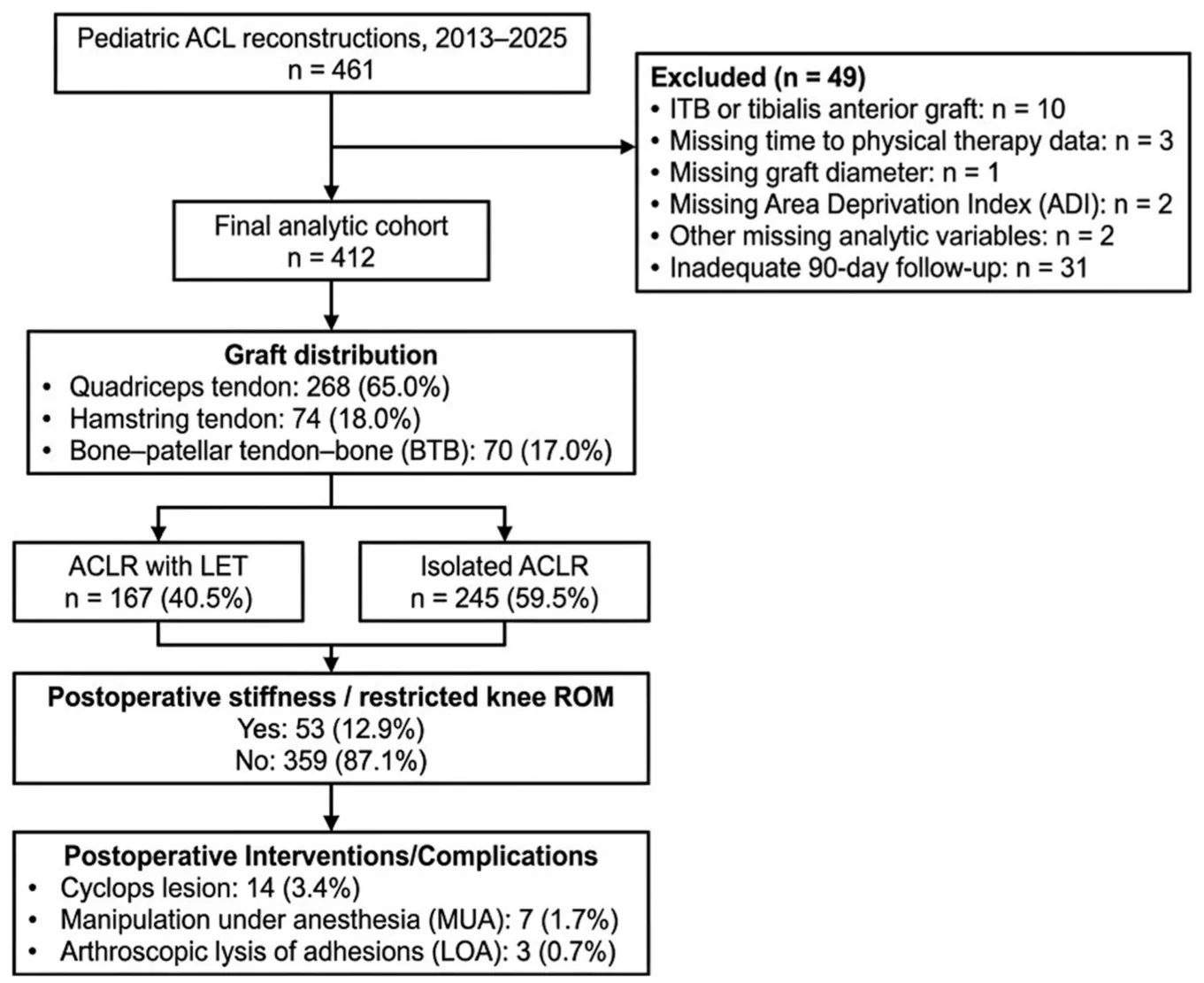
Study cohort flow diagram. ACLR, anterior cruciate ligament reconstruction; ADI, Area Deprivation Index; LET, lateral extra-articular tenodesis; MUA, manipulation under anesthesia.

**Table 1:** Baseline Patient and Clinical Characteristics of the Study Population^*a*^

Characteristic	Overall n = 412 procedures, 406 patients
Patient characteristics	
Age at surgery, years	16.3 ± 1.4
Female sex	199 (48.3)
Insurance type	
Medicaid	301 (73.1)
Commercial, HMO/PPO	111 (26.9)
Socioeconomic status	
ADI state decile	5.4 ± 2.3
High deprivation, ADI ≥ 6	215 (52.2)
ACL rupture laterality	
Left knee	212 (51.5)
Right knee	200 (48.5)
Meniscal procedures	
Lateral meniscus repair	89 (21.6)
Lateral meniscus debridement	87 (21.1)
Medial meniscal repair	55 (13.3)
Medial meniscal debridement	27 (6.6)
Graft diameter	9 (6-11)
Radiographic measurements	
Notch width, mm	19.5 ± 2.6
Male notch width, mm	20.8 ± 2.5
Female notch width, mm	18.2 ± 2.1
Concomitant procedures	
LET performed	167 (40.5)

a Data are presented as mean ± SD, median (range), or n (%). ACL, anterior cruciate ligament; ADI, Area Deprivation Index; HMO, health maintenance organization; LET, lateral extra-articular tenodesis; PPO, preferred provider organization.

A total of 461 pediatric ACLRs performed between 2013 and 2025 were identified. Of those, 49 cases were excluded after applying the inclusion criteria. The final analytic cohort consisted of 412 primary ACLRs utilizing quadriceps tendon, hamstring tendon, or BPTB autografts. LET augmentation was performed in 167 cases (40.5%). Restricted knee ROM occurred in 53 patients (12.9%) at 3 months postoperatively.

### Surgical Technique and Perioperative Details

Most procedures utilized quadriceps tendon autograft (n = 268; 65%), followed by hamstring tendon (n = 74; 18%) and BPTB (n = 70; 17%) grafts. Graft distribution differed significantly by LET status. Quadriceps tendon autograft was used in 139 of 167 LET cases (83.2%) compared with 129 of 245 non-LET cases (52.7%), while BPTB autograft was used only in the non-LET group (*P* < .001). The graft diameter had a median of 9 mm (range, 6-11 mm). Radiographic notch width averaged 19.5 ± 2.6 mm overall, with significantly larger notches in males (20.8 ± 2.5 mm) compared with females (18.2 ± 2.1 mm; *P* < .001). Medial meniscal repair was performed in 13.3% (n = 55) of cases and debridement in 6.6% (n = 27), while lateral meniscal repair and debridement were performed in 21.6% (n = 89) and 21.1% (n = 87) of cases, respectively. LET was performed in 40.5% (n = 167) of procedures (Table 1).

### Functional Outcomes and Time to Physical Therapy

Restricted knee ROM occurred in 28 of 167 procedures (16.8%) after ACLR with LET, compared with 25 of 245 procedures (10.2%) after isolated ACLR. Overall, restricted knee ROM occurred in 12.9% of procedures (n = 53), including 13 males (24.5%). Among the 53 patients meeting criteria for 3-month postoperative restricted ROM, 11 had an isolated extension deficit (20.8%), 34 had an isolated flexion deficit (64.1%), and 8 had combined flexion and extension deficits (15.1%). Deficit pattern did not differ significantly by LET status (Fisher exact *P* = .567). Among non-LET patients with restricted ROM, 6 had extension-only deficits, 14 had flexion-only deficits, and 5 had combined deficits. Among LET patients with restricted ROM, 5 had extension-only deficits, 19 had flexion-only deficits, and 3 had combined deficits. Postoperative restricted ROM did not significantly differ by graft type, occurring in 38 of 268 quadriceps tendon grafts (14.2%), 9 of 74 hamstring tendon grafts (12.2%), and 6 of 70 BPTB grafts (8.6%; *P* = .450). A cyclops lesion was identified in 3.4% of patients (n = 14). Procedural intervention for postoperative restricted knee ROM was uncommon but differed by LET status. MUA was required in 7 patients overall and was more frequent among LET patients than non-LET patients (6/167 [3.6%] vs 1/245 [0.4%]; Fisher exact *P* = .019). The mean time to MUA was 128.6 days, with procedures occurring between 91 and 160 days postoperatively. LOA was required in 3 patients, all of whom underwent LET (3/167 [1.8%] vs 0/245 [0%]; Fisher exact *P* = .066). The mean time to LOA was 370.7 days. However, this was influenced by 1 late procedure at 808 days, with the remaining procedures occurring at 144 and 160 days. Overall, MUA or LOA was required in 8 patients and was more common after LET (7/167 [4.2%] vs 1/245 [0.4%]; Fisher exact *P* = .009), suggesting that LET was associated not only with increased early ROM restriction but also with higher rates of subsequent procedurally treated arthrofibrosis. Initiation of formal physical therapy occurred within 13 days of surgery in most cases (n = 281; 68.2%), between 14 and 30 days in 78 cases (18.9%), and >30 days in 53 cases (12.9%). The mean state-level ADI did not differ significantly by time to physical therapy (<14 days: 5.2 ± 2.3;14-30 days: 5.7 ± 2.4; >30 days: 5.7 ± 2.2; *P* = .170) (Table 2).

**Table 2:** Early Postoperative Complications and Physical Therapy Initiation Timing by ADI^*a*^

Category	Variable	Percentage	Cohort, N	Additional Details, n (%)
Complications	Restricted knee ROM	12.9	53	13 (24.5) male
	Cyclops lesions	3.4	14	
	Required MUA	1.7	7	LET: 6/167 (3.6) vs non-LET: 1/245 (0.4); *P* = .019
	Required LOA	0.7	3	LET: 3/167 (1.8) vs non-LET: 0/245 (0); *P* = .066
	Required MUA or LOA	1.9	8	LET: 7/167 (4.2) vs non-LET: 1/245 (0.4); *P* = .009
PT initiation	Within 13 days	68.2	281	Mean ADI 5.2 ± 2.3
	14-30 days	18.9	78	Mean ADI 5.7 ± 2.4
	>30 days	12.9	53	Mean ADI 5.7 ± 2.2

a Data are presented as n, n (%), or mean ± SD. ADI, area deprivation index; LET, lateral extra-articular tenodesis; LOA, lysis of adhesions; MUA, manipulation under anesthesia; PT, physical therapy; ROM, range of motion.

### Factors Associated with Restricted Knee ROM

Female sex was independently associated with significantly increased odds of restricted knee ROM (AOR, 4.23 [95% CI, 2.09-8.55]; *P* < .001), as was LET utilization (AOR, 2 [95% CI, 1.01-3.95]; *P* = .046). Age at surgery demonstrated a protective association that did not reach statistical significance after adjustment for time from injury to surgery (AOR, 0.80 [95% CI, 0.63-1.02]; *P* = .073). Time to physical therapy initiation was not associated with restricted knee ROM (14-30 days vs <14 days: AOR, 0.85 [95% CI, 0.36-1.99]; *P* = .702; >30 days vs <14 days: AOR, 1.37 [95% CI, 0.54-3.48]; *P* = .509). Meniscal procedures were not significant predictors (AOR, 1.39 [95% CI, 0.73-2.63]; *P* = .314). Graft type was not associated with restricted knee ROM, including hamstring tendon autograft compared with quadriceps tendon autograft (AOR, 0.68 [95% CI, 0.27-1.74]; *P* = .424) or BPTB autograft compared with quadriceps tendon autograft (AOR, 1.07 [95% CI, 0.37-3.06]; *P* = .905). Time from injury to surgery was not associated with restricted knee ROM (AOR, 1 [95% CI, 1-1]; *P* = .724). Race, insurance status, and state-level ADI were also not significantly associated with restricted knee ROM (Figure 2).

**Figure 2.**
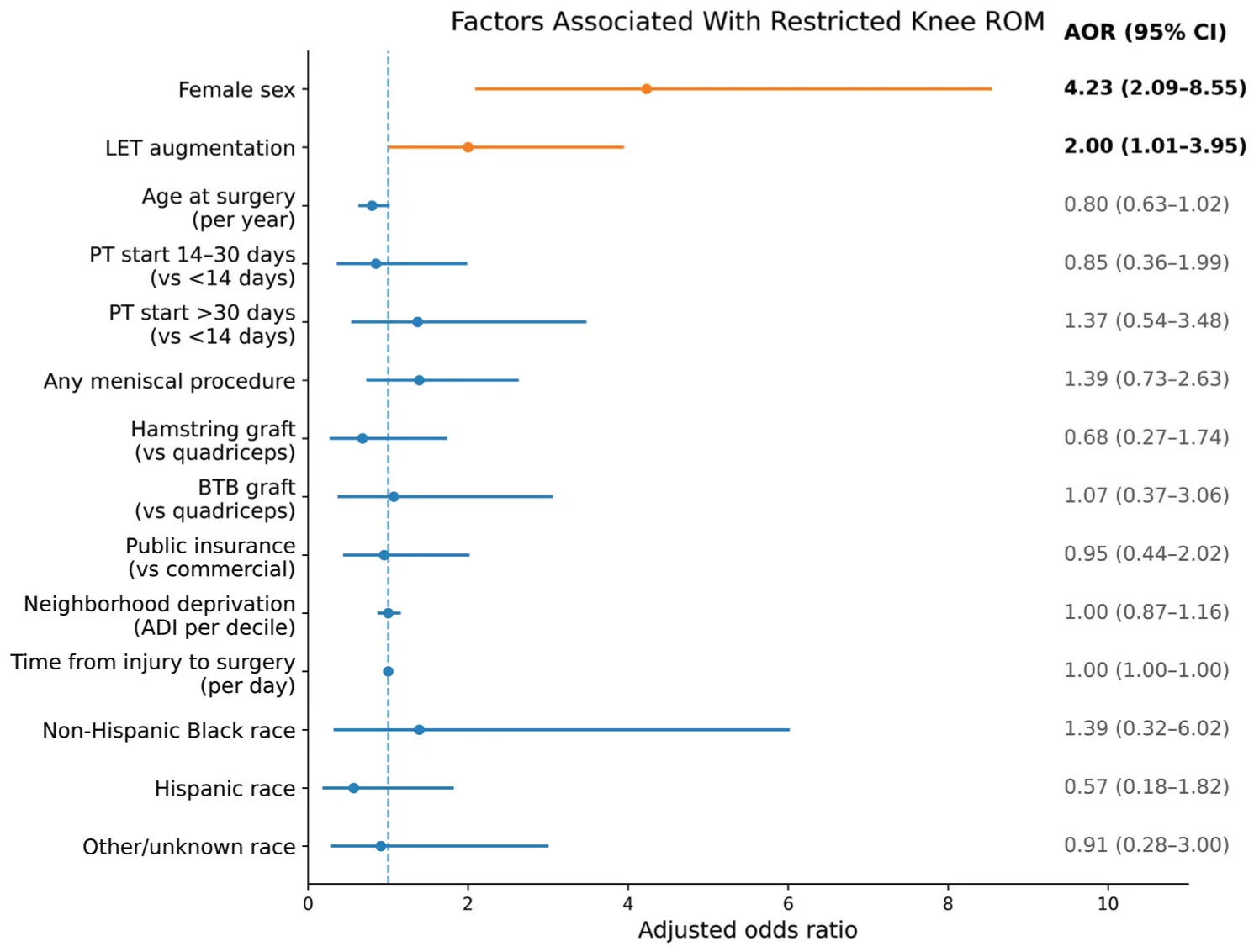
Predictors of restricted knee ROM after ACLR. Forest plot showing odds ratios and 95% CIs from multivariable logistic regression. Orange indicates *P* < .05; Blue indicates nonsignificant findings. The dashed vertical line represents OR = 1. ACLR, anterior cruciate ligament reconstruction; OR, odds ratio; ROM, range of motion.

## Discussion

This study aimed to identify risk factors for early restricted knee ROM in a large, single-center pediatric cohort. Our findings suggest that LET augmentation during primary ACLR is independently associated with an increased risk of restricted knee ROM at 3 months postoperatively. After adjustment for patient and surgical factors, LET augmentation increased the risk of postoperative restricted knee ROM at 3 months compared with isolated ACLR. Likewise, male sex was protective against postoperative stiffness. Graft diameter, meniscal procedures, socioeconomic deprivation, time to initiation of physical therapy, and graft type were not independently associated with restricted motion. These findings suggest that while LET may reduce the risk of graft failure, it carries a measurable trade-off in early postoperative knee mobility in pediatric patients.

LET augmentation during ACLR provides additional load sharing, reducing ACL graft forces by up to 80% and decreasing anterior tibial translation in the lateral compartment. LET also appears to mitigate the increase in graft forces associated with greater posterior tibial slope.^19^ These biomechanical advantages likely contribute to the lower graft-failure rates reported in contemporary combined ACLR and LEAP series. Although the STABILITY trial provided high-level evidence confirming the graft-protective effect of LET in young high-risk patients, the renewed adoption of lateral extra-articular augmentation was supported by earlier European work demonstrating improved rotatory stability and favorable clinical outcomes after combined intra- and extra-articular reconstruction.^9,11,35^ Consistent with this trend, 2024 international consensus guidelines recommend LET in young, active patients undergoing hamstring tendon autograft ACLR; skeletally immature patients; and in cases involving high-grade rotational or anterior laxity, knee hyperextension, revision surgery, return to pivoting sports, posterior tibial slope >12°, or a history of contralateral ACL injury.^28,31^ LET should also be considered in adolescents undergoing patellar tendon or quadriceps autograft ACLR.^28^

However, the expanding use of LET should still be viewed in light of its historical background. Extra-articular reconstructions were widely used in earlier eras of ACL surgery but declined after concerns regarding morbidity, overconstraint, lateral compartment degeneration, and inconsistent clinical benefit, concerns that were amplified by open techniques, frequent BPTB graft use, postoperative immobilization, and delayed rehabilitation.^30^ Contemporary LEAPs have been reintroduced in a different surgical and rehabilitation environment, with arthroscopic ACLR, more selective indications for high-risk rotatory instability, less invasive lateral procedures, and accelerated rehabilitation protocols emphasizing early motion.^28^ Ferretti et al and subsequent long-term European series helped reframe the role of combined intra- and extra-articular reconstruction, demonstrating durable stability without clear evidence that lateral extra-articular augmentation inevitably produces lateral or patellofemoral osteoarthritis.^9,13^ In pediatric ACLR, the relevant question is therefore not simply whether LET can reduce graft failure, but how that benefit should be weighed against early postoperative morbidity. The present findings support that risk-benefit framing: LET was associated with increased ROM-based postoperative stiffness and higher rates of MUA/LOA, although most stiffness cases resolved without procedural intervention. These findings reinforce the importance of careful patient selection, meticulous LET technique, avoidance of overconstraint, early supervised rehabilitation, and close postoperative monitoring of ROM recovery.

Although concerns regarding stiffness, arthrofibrosis, and overconstraint have long shaped the historical use of extra-articular procedures, contemporary pediatric data evaluating ROM-based postoperative stiffness after ACLR with LET remain limited. This distinction is important because modern LET is performed in the setting of arthroscopic ACLR, less invasive lateral extra-articular techniques, and early-motion rehabilitation protocols, rather than the more morbid open techniques and prolonged immobilization or delayed-motion protocols that contributed to the decline of extra-articular procedures in earlier eras. Previous multicenter and single-surgeon series have reported low absolute rates of stiffness requiring intervention after LET, generally ranging from 1% to 2%.^7,27^ DeFrancesco et al evaluated 569 patients undergoing hamstring tendon autograft ACLR with or without LET augmentation and found no difference in arthrofibrosis rates.^7^ However, this single-surgeon series routinely incorporated LET only during the final 2 years of the study period and did not include extensor mechanism autografts.^7^ Importantly, these studies largely relied on procedural endpoints such as MUA or LOA, thereby capturing arthrofibrosis requiring procedural treatment rather than the broader spectrum of ROM-based postoperative motion restriction that may affect rehabilitation progress and patient experience. For this reason, we analyzed MUA and LOA separately as procedural arthrofibrosis outcomes, while retaining 3-month-restricted knee ROM as the primary postoperative stiffness endpoint. Previous pediatric ACLR studies without routine LET augmentation have reported arthrofibrosis or stiffness rates ranging from approximately 1.8% to 8.3%, including an 8.3% rate in the series by Nwachukwu et al^25^ and graft-specific rates reported by Ouweleen et al,^26^ with the quadriceps tendon autograft associated with a 6.3% arthrofibrosis rate. In this context, the 10.2% restricted-ROM rate in the non-LET cohort is near the upper range of published pediatric ACLR stiffness estimates. In contrast, the 16.8% rate after LET suggests an increased burden of early motion restriction. These comparisons should be interpreted with caution, however, given that our study’s primary endpoint was a 3-month ROM-based measure rather than a procedural or persistent-stiffness endpoint.

The demonstrated association between LET and early restricted knee ROM has important implications for patient counseling and rehabilitation after pediatric ACLR. Importantly, the primary endpoint in this study reflects restricted knee ROM at the 3-month postoperative visit rather than established long-term arthrofibrosis. Most cases of early restricted ROM resolved with continued rehabilitation and did not require procedural intervention, whereas a small subset progressed to arthrofibrosis requiring MUA or LOA. These procedures were more frequent in the LET cohort than in the non-LET cohort, suggesting that LET-associated stiffness included both early motion restriction and a smaller group with procedurally treated arthrofibrosis. Among patients requiring procedural treatment, 2 underwent both MUA and LOA. Both patients had undergone LET, further suggesting that the more refractory cases of postoperative arthrofibrosis occurred within the LET cohort. Given that LET augmentation is increasingly being used in high-risk pivoting athletes, surgeons should weigh the benefit of graft protection against the increased risk of early stiffness, including the small subset of patients who may progress to procedurally treated arthrofibrosis. Considerations include early motion protocols in patients undergoing augmentation. In addition, future biomechanical studies investigating LET tunnel placement and standardized graft tensioning may also provide additional insight into techniques to improve more natural knee kinematics.^34^

Socioeconomic context may also affect how postoperative stiffness is recognized, treated, and compared across pediatric ACLR cohorts. Our cohort included a high proportion of publicly insured patients and a higher mean state-level ADI, reflecting a more socioeconomically disadvantaged population than previous sports medicine series. Although neither ADI nor insurance status was independently associated with postoperative stiffness in the present multivariable model, these variables are imperfect proxies for the practical barriers that often shape early recovery, including transportation reliability, therapy authorization, caregiver work constraints, distance from high-quality rehabilitation, and consistency of postoperative follow-up. Accordingly, differences in socioeconomic composition and access to rehabilitation should be considered when comparing restricted ROM rates across LET cohorts.

Similar to other pediatric cohorts, male sex was independently protective against postoperative restricted knee motion.^25,26^ It is unknown why this observed phenomenon occurs, especially in pediatric populations. It is hypothesized that sex-related differences in intra-articular inflammatory responses may contribute to excessive scar formation, especially in female pediatric patients with larger grafts.^2,12^ Sex-specific differences in knee morphology, graft size relative to notch dimensions, pain-mediated quadriceps inhibition, and neuromuscular recovery may also contribute to delayed restoration of terminal motion. Injury chronicity may also be relevant to this inflammatory framework, because acute or subacute ACLR may occur in a more reactive intra-articular environment. For this reason, time from injury to surgery was added to the multivariable model. In the present study, injury-to-surgery interval was not independently associated with restricted knee ROM, and the association between LET and restricted motion remained significant after adjustment. Although this reduces concern that injury chronicity was the primary driver of the LET association, residual confounding related to injury acuity remains possible. Taken together, these findings suggest that risk for postoperative restricted ROM is likely multifactorial, reflecting patient-level factors, surgical augmentation, and the perioperative inflammatory environment. Therefore, in pediatric patients undergoing ACLR with LET, sex and injury chronicity should remain important considerations during counseling and postoperative ROM surveillance.

This study has several important strengths, including a relatively large pediatric cohort, inclusion of multiple graft types, and detailed adjustment for patient, surgical, rehabilitation, and socioeconomic factors. These features enhance the clinical relevance of our findings and distinguish this cohort from prior studies that were limited to narrower graft populations and smaller pediatric samples. However, several limitations should also be considered. The retrospective, single-institution design at a tertiary-care, urban, academic children’s hospital may limit generalizability. ROM was assessed only at routine postoperative intervals, and the long-term functional implications of early stiffness were not evaluated. Additionally, ROM measurements were obtained retrospectively from routine clinical documentation rather than by independent evaluators using a prospective standardized goniometric protocol, which may introduce measurement variability. Technical heterogeneity also existed across surgeons and operative eras. ACL graft choice, tunnel technique, and fixation construct were individualized according to surgeon preference and patient-specific factors, while LET fixation evolved and included both interference screw and knotless all-suture anchor fixation. Although ACL grafts were generally tensioned in full extension and LET grafts were fixed at 30° to 45° of knee flexion with neutral rotation, granular technical variables were not uniformly documented in a standardized format, limiting the ability to evaluate surgeon- or technique-specific associations with postoperative stiffness. Although formal physical therapy was prescribed for all patients and ROM progression was not intentionally restricted based on LET status, patient-level differences in rehabilitation adherence, therapy frequency, and progression could not be fully captured retrospectively. Finally, although we adjusted for key confounders using multivariable modeling, residual confounding by indication remains possible, as patients selected for LET augmentation may represent a higher-risk instability subgroup despite adjustment for measured patient and surgical factors. Unmeasured factors, such as postoperative pain control strategies and variation in physical therapy protocols, may also have influenced outcomes, as these data were unavailable.

## Conclusion

In a large single-institution pediatric cohort undergoing primary ACLR, LET augmentation was associated with increased odds of 3-month postoperative restricted knee ROM compared with isolated ACLR. Although most motion deficits were managed nonoperatively, this finding highlights an important early postoperative tradeoff associated with LET augmentation rather than definitive evidence of clinically persistent arthrofibrosis. Male sex was protective against postoperative stiffness, whereas graft type, meniscal procedures, socioeconomic factors, time from injury to surgery, and time to physical therapy initiation were not independently associated with restricted ROM. As LET use continues to expand in high-risk pediatric athletes, these results underscore the importance of careful patient selection, counseling, and postoperative rehabilitation strategies. Future prospective studies are needed to determine whether early motion deficits affect long-term functional outcomes and to identify surgical or rehabilitation approaches that mitigate the risk of stiffness while preserving the graft-protective benefits of LET.
